# Screening and characterization of cellulolytic molds from empty fruit bunches and soils in palm oil plantation area in Indonesia

**DOI:** 10.1186/s13104-021-05668-8

**Published:** 2021-06-30

**Authors:** Tracy Miller, Diana Elizabeth Waturangi, Tresnawati Purwadaria

**Affiliations:** Faculty of Biotechnology, Atma Jaya Catholic University of Indonesia, Jalan Jenderal Sudirman 51, Jakarta, 12930 Indonesia

**Keywords:** Empty fruit bunches of oil palm, Cellulolytic molds, Enzyme activity

## Abstract

**Objective:**

This research was aimed to isolate cellulolytic molds in empty fruit bunches of oil palm (EFBOP) and soils from palm oil plantation area and identify their enzyme activities to digest EFBOP.

**Results:**

A total of seven molds were successfully isolated and screened for their enzyme activities from EFBOP and the soils. The enzymes from each isolate were produced in submerged culture using Mineral Mandels and 3% of alkali pretreated pollard in triplicates. The results indicated that all of the isolates were able to hydrolyze Carboxymethyl Cellulose (CMC), Whatmann No. 1 filter paper, and also EFBOP to sugars with reducing ends that reacted to 3,5-Dinitrosalicylic acid (DNS). The CMCase activity of isolate X showed the highest while the lowest was found for isolate MT8. Filter paperase (FPase) activity of isolate X performed the highest wile the lowest were found from isolate MT3 and MT6. The saccharification activity of isolate P showed the highest while MT6 performed the lowest activity

**Supplementary Information:**

The online version contains supplementary material available at 10.1186/s13104-021-05668-8.

## Introduction

Empty fruit bunches of oil palm (EFBOP) is one of the solid wastes produced from CPO (crude palm oil) mills which have not been utilized commercially. Currently, its use is only limited as fuel at the CPO mill itself or uses as a compost in oil palm plantations. These wastes are potentially to be recycled and to produce synthetic biofuel for power generation, bioethanol, as well as biocomposite product from the cellulose and lignin [1]. On the other hand, EFBOP is also a substrate for the growth of variant molds, such as *Trichoderma*, *Aspergillus*, *Mucor*, *Penicillium*, and *Neurospora crassa* [2, 3]. These cellulolytic molds can hydrolyze cellulose fibrous materials into oligosaccharides, cellobiose, and glucose.

There has been a notable increase in interest about cellulases in recent years due to their numerous potential applications, including the hydrolysis of cellulose in lignocellulosic biomass to produce reducing sugars [4]. Application of the cellulase may reduce the amount of the waste. Therefore, research to explore microorganisms capable in degrading several major components of oil palm need to be conducted. The aim of this study is to isolate cellulolytic molds in EFBOP and soils from palm oil plantation area for identify their enzyme activities to digest EFBOP.

## Main text

### Methods

#### Isolation of cellulolytic molds in EFBOP and soil

Firstly, EFBOP were cut about 1 cm. The milled bunches were enriched with 10% (w/v) Buffered Peptone Water, then incubated in waterbath shaker for 2 h at 100 rpm in room temperature. Then, a mixture consisting of EFBOP and media was filtered using filter cloth and 25 µL of filtrates were spreaded onto Potato Dextrose agar (PDA) and incubated for 3 days at 30 °C [5]. Meanwhile, one gram of soil samples were serially diluted until reached 10^–3^ concentration in sterile physiological solutions (0.85% of NaCl) (w/v). Then, 100 µL of each suspensions was spreaded onto PDA and incubated at 30 °C for 3 days. PDA were supplemented with chloramphenicol (0.05 g/L) to prevent growth of bacteria. The growing mold was then purified and subcultured in the new PDA plate [6].

#### Production of cellulase

The enzyme was produced in submerged culture using 50 mL of Mandel’s medium, then added with 0.05% of yeast extract, 0.075% of peptone, and 3% of alkali pretreated pollard. The spores were determined to reach 10^9^ spores/mL using hemacytometer and then 2 ml of spores suspension were inoculated into each flasks. Incubation was carried out on a waterbath shaker at 150 rpm at 30 °C for 3–5 days. At the time of harvesting, culture was added with 0.2% of Na-azide. The supernatant were used as a source of extracellular enzymes. Enzyme extract was stored in a freezer (− 20 °C) for further analysis [7].

#### EFBOP and pollard alkali pretreatment

EFBOP or pollard (100 g) was mixed in 500 mL of 0.5% of NaOH and then boiled at 100 °C for 2 h. After the process was completed, a mixture consisting of lignocellulosic biomass and NaOH was filtered to separate the EFBOP solid and black liquor containing lignin. The filtered EFBOP or pollard was washed with running tap water until neutral pH, and then dried in an oven at 50 °C for 48 h and ground to become powder. Pretreated EFBOP was used in saccharification activity and pretreated pollard was used in production of cellulase [8].

#### Carboxymethylcellulase (CMCase) and filterpaperase (FPase) activity

CMCase activity was assayed by determining the reducing sugars produced from CMC as glucose or mannose. For the control of reaction mixture, the enzyme activity was inactivated by the addition of DNS reagent before the substrate addition and incubation. One unit of enzyme activity was defined as the amount of enzyme that will produce reducing sugar equal to 1 µmol of glucose per minute in assay condition [6, 9]. FPase activity was determined by using filter paper Whatman no 1 (1 cm × 6 cm) as a substrate. Control was prepared with a mixture of 0.5 mL of sodium citrate buffer, 1.5 mL of DNS, and 0.5 mL of enzyme. One unit of this activity was defined as the amount of enzyme that liberated 1 mM of reducing sugars per minute under assay condition [4, 9].

#### Saccharification activity

Saccharification activity was carried out using 1% of alkali pretreated EFBOP as the substrate in sodium acetate buffer (0.1 M, pH 4.8). The optimum incubation time of saccharification activity was firstly determined for 2, 4, 6, and 7 h. Reducing sugar produced by saccharification activity was determined with DNS method [10] at pH 4.8, and temperature at 50 °C. Control was prepared with same treatment as sample, but boiled for 10 min then added with alkali pretreated EFBOP and boiled for 10 min. The activity is expressed in 1 µmol of glucose produced per minute under assay condition [9].

#### Protein content determination

Protein concentration was determined by Bradford method [11]. As much as 50 µL of sample was added with 2.5 mL of Coomassie G-250. Bovine Serum Albumin was used as standard. The absorbance was measured at λ 595 nm. Specific activity of CMCase, FPase, and saccharification were measured [12].

#### Statistical analysis

The significance of the data was evaluated using one-way analysis of variance ANOVA. All of the analysis were performed with Statistical Package for the Social Sciences (SPSS) version 25. Statistical analysis was performed in a one-way complete randomized design with 7 isolates and 3 replications [13].

#### PCR amplification of the ribosomal internal transcribed spacer and sequencing

Mold isolates were cultured using PDA medium for three days, then DNA of mold were extracted using ZymoBIOMICS DNA miniprep kit (Zymo Research). Isolates were identified by amplification and DNA sequencing of the ribosomal transcribed spacer (ITS) region using ITS5 (5ʹ-GGAAGTAAAAGTCGTAACAAGG-3ʹ) and ITS4 (5ʹ-TCCTCCGCTTATTGATATGC-3ʹ) primers [14].

### Results

#### Isolation of molds from EFBOP and soil

**Fig. 1:**
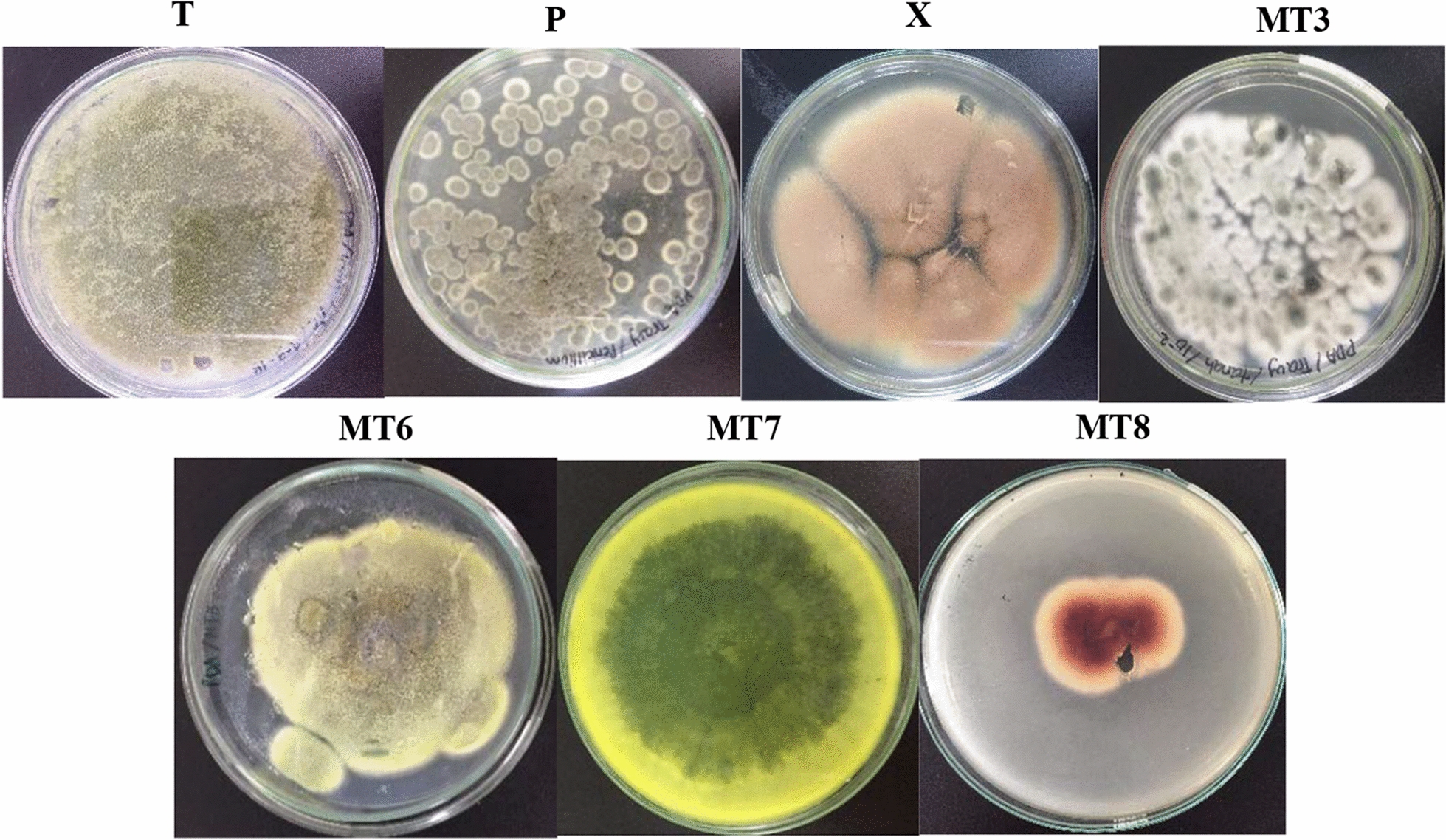
Results of mold isolation from EFBOP (T, P, X) and soils (MT3, MT6, MT7, MT8) in palm oil plantation area

Seven isolates of mold consisting of 3 isolates (T, P, and X) from EFBOP and 4 isolates (MT3, MT6, MT7, and MT8) from soil around EFBOP were successfully recovered (Fig. 1). Macroscopic observations of mold isolates were determined which included several parameters, including color of the colonies, surface/texture, reverse colonies, and the growth. The results of morphological observations of isolate T showed several macroscopic characteristics such as powdery light green, full growth and spread over on Petri-dish at day 2–4; isolate P showed rapid growing, round shape, velvety, green; while isolate X performed powdery, beige to buff to cinnamon, moderate to rapid growth rate, finely granular; isolate MT3 had woolly to somewhat granular texture, the color was initially white and became green at the top of colonies; isolate MT6 showed powdery, light green, rapid growing, and velvety; isolate MT7 was powdery, dark green, reverse was yellow; and the last is isolate MT8 showed initially white and became red, woolly, and slow growth.

#### Enzyme activities

These isolates showed various CMCase, FPase, and saccharification activities (Table 1). Regarding to the CMCase activity, isolate X performed the highest enzyme activity (0.46 ± 0.02 U/mL) and isolate MT8 showed the lowest activity (0.08 ± 0.01 U/mL). Regarding to the FPase and saccharification activity, isolate T, P, and X displayed the highest activity while isolate MT6 showed the lowest activity.

**Table 1:** Enzyme activities from isolates collection

Isolate	CMCase activity (U/mL)^a^	FPase activity (U/mL)^a^	Saccharification activity (mU/mL)^a^
T	0.24 ± 0.02	0.28 ± 0.05	3.52 ± 1.39
P	0.13 ± 0.06	0.28 ± 0.05	3.81 ± 0.72
X	0.46 ± 0.02	0.29 ± 0.07	2.45 ± 1.10
MT3	0.10 ± 0.03	0.13 ± 0.02	2.42 ± 1.52
MT6	0.14 ± 0.06	0.13 ± 0.01	0.78 ± 0.53
MT7	0.20 ± 0.09	0.27 ± 0.05	0.96 ± 0.20
MT8	0.08 ± 0.01	0.24 ± 0.05	2.21 ± 1.29
p	0.16	0.06	0.09

p was probability

^a^CMCase ± standard error, FPase ± standard error, Saccharification ± standard error

#### Protein content and specific activities determination

Protein content and specific activities of CMCase, FPase, as well as saccharification were shown in Table 2. The result showed that isolate MT8 had the lowest CMCase activity (0.08 ± 0.01 U/mL) but it had highest protein content (182.56 ± 35.44 mg/mL) producing relatively low specific activity value. However, isolate X showed the highest activity (0.46 ± 0.02 U/mL) but the protein content quietly low (121.44 ± 5.98 mg/mL), it produced high specific activity value. It was known that the protein value will correlate positively with enzyme activity, but our data not following this term.

**Table 2:** Protein content and specific activities of CMCase, FPase, and saccharification

Isolate	Protein content (mg/mL)^a^	Specific activity^a^		
		CMCase (U/mg protein)	FPase (U/mg protein)	Saccharification (mU/mg protein)
T	125.93 ± 8.26	0.19 ± 0.02	0.23 ± 0.06	2.80 ± 1.07
P	127.85 ± 15.47	0.10 ± 0.03	0.22 ± 0.04	3.05 ± 0.68
X	121.44 ± 5.98	0.38 ± 0.12	0.25 ± 0.08	1.94 ± 0.85
MT3	67.90 ± 19.55	0.15 ± 0.04	0.23 ± 0.07	2.52 ± 1.21
MT6	81.21 ± 13.01	0.16 ± 0.06	0.17 ± 0.02	0.81 ± 0.46
MT7	106.62 ± 23.00	0.21 ± 0.10	0.30 ± 0.10	1.00 ± 0.29
MT8	182.56 ± 35.44	0.04 ± 0.01	0.15 ± 0.04	1.29 ± 0.72
p	0.06	0.07	0.15	0.10

p was probability

^a^Protein content/specific activity ± standard error

#### PCR amplification of the ribosomal internal transcribed spacer and sequencing

DNA sequencing analysis showed that each of the isolates T, P, X, MT3, MT6, MT7, and MT8 respectively showed similarities with *Aspergillus flavus* (accession number MW513940), *Penicillium citrinum* (MW513937), *A. alabamensis* (MW513987), *Talaromyces thailandensis* (MW514041), *T. rufus* (MW514039), *Trichoderma reesei* (MW514156), and *Monascus pilosus* (MW514155). All of these isolates had a similarity of more than 95% except for MT3 isolates which had a similarity of 85%. All of the data have been submitted to Genbank with the accession number informed above.

### Discussion

All of the isolates obtained in this study were quite diverse in terms of phenotypic properties such as color, shape, and texture. However, the mold isolates recovered from EFBOP is limited due to EFBOP samples were dry and not moldy. Therefore, mold isolates obtained from the soil were slightly more than those obtained from EFBOP due to the humidity of the soils were higher compare with EFBOP.

Regarding to the CMCase activity, isolate X gave the highest CMCase activity. Isolate X displayed the highest CMCase activity (0.46 U/mL) compared with the activity of isolate MT8 which was only 0.08 U/mL. The average difference of CMCase between isolate X and MT8 was 5× fold (Table 1).

Respect to the FPase activity, isolate X recorded the highest value of total FPase activity (0.29 U/mL) compared with activity value of 0.13 U/mL produced by MT3 and MT6 with an average difference of 2× fold (Table 1). Some of FPase activity showed higher than CMCase activity, the reason is due to the molds in this study were recovered from natural environment containing high crystalline cellulose such as in EFBOP and the soil around the palm oil contain humus from the plant [9].

Regarding to the saccharification activity, isolate P performed the highest activity and the lowest was found from isolate MT6. The difference in average saccharification between P and MT6 isolates was 4× fold (Table 1). The saccharification activity was related to CMCase and FPase activities. Although the CMCase activity of all mold isolates were not very high compared to the FPase activity, all of mold isolates were still able to efficiently convert pretreated EFBOP to fermentable sugars.

Pre-treatment of EFBOP and pollard by milling increased the ratio of surface area to volume resulting to better mold growth and enzyme production. Meanwhile, alkali pretreatment using NaOH solution can remove or modify its lignin by fracturing the ester bonds that form cross-links between xylan and lignin, thereby increasing the porosity of biomass [9, 15].

Regarding to specific activities for CMCase, FPase, and saccharification, isolate MT8 showed the lowest CMCase activity but the highest protein content producing relatively low specific activity value. However, isolate X showed the highest CMCase activity but the protein content quietly low producing high specific activity value (Table 2). It was known that the protein value will correlate positively with enzyme activity, but in our data some of specific activity values are not affected by protein concentration. If the protein content was increased, the enzyme activity should increase. However, there were some isolates which did not show this phenomena. If the protein content was high, but the enzyme activity was low, it might indicated that the enzymes contained a lot of impurities so the enzyme activity was less specific [9].

PCR amplification was done after the DNA extraction process was successfully completed. Two pairs of primers (ITS5-F and ITS4-R) were used. DNA ladder 1 kb was used as a marker. The size of the PCR products is approximately 534–647 bp (see Additional file 1). DNA sequencing analysis showed that two isolates belong to the genus of *Aspergillus*, two isolates belong to the genus of *Talaromyces*, and the others belong to the genus of *Penicillium*, *Trichoderma*, and *Monascus*.

### Conclusion

Cellulolytic molds were successfully isolated from EFBOP and soil in palm oil plantation area. In this research, three potential mold isolates were obtained and showed activities in degrading EFBOP. The highest CMCase value was obtained from *Aspergillus alabamensis*. The highest FPase and saccharification activity was obtained from *Aspergillus flavus, Penicillium citrinum*, and *Aspergillus alabamensis*. Therefore, *Aspergillus flavus*,* Penicillium citrinum*, and *Aspergillus alabamensis* have the potential as EFBOP degradation agents.

## Limitations

Activity of CMCase, FPase, and saccharification might be different in different substrate due to different lignocellulose compounds. Statistical analysis of all enzyme activities stated that there were no significant difference due to high variability between replicates. Optimization of the substrate and microenvironment (temperature and pH) for each species of mold and optimization of EFBOP pre-treatment has not been done. Application of molds in the composting process in a pile of EFBOP needs further evaluation.

## Supplementary Information

**Additional file 1. **Visualization of fungus DNA products from EFBOP (1,2,3) and soils (4, 5, 6, 7) on agarose gel 1.2%: (1) 595 bp, (2) 534 bp, (3) 600 bp, (4) 560 bp, (5) 594 bp, (6) 647 bp, (7) 548 bp.

## Data Availability

The datasets used and/or analysed during the current study are available from the corresponding author on reasonable request.

## Abbreviations

EFBOP: Empty fruit bunches of oil palm
CMCase: Carboxymethylcellulase
FPase: Filterpaperase
