# Supplementary figures and images for: Screening and characterization of cellulolytic molds from empty fruit bunches and soils in palm oil plantation area in Indonesia
Source: BMC Res Notes. 2021 Jun 30;14:249. doi: 10.1186/s13104-021-05668-8 (PMC8244140; doi:10.1186/s13104-021-05668-8)

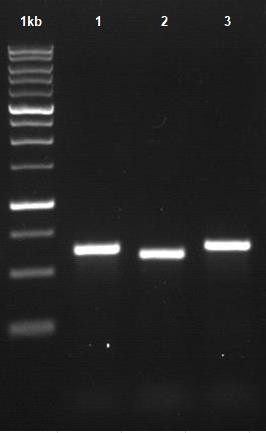

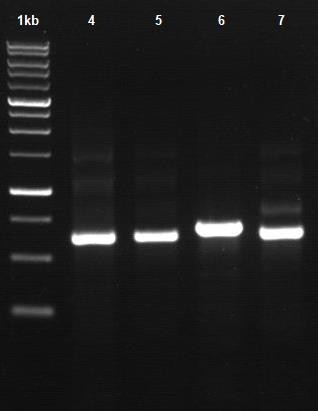


Visualization of fungus DNA products from EFBOP (1,2,3) and soils (4, 5, 6, 7) on agarose gel 1.2%: (1) 595 bp, (2) 534 bp, (3) 600 bp, (4) 560 bp, (5) 594 bp, (6) 647 bp, (7) 548 bp.

Supplement: Supplementary file 1 — Additional file 1. Visualization of fungus DNA products from EFBOP (1,2,3) and soils (4, 5, 6, 7) on agarose gel 1.2%: (1) 595 bp, (2) 534 bp, (3) 600 bp, (4) 560 bp, (5) 594 bp, (6) 647 bp, (7) 548 bp. [file 13104_2021_5668_MOESM1_ESM.docx]
